# Supplementary material for: Chromium Methionine and Ractopamine Supplementation in Summer Diets for Grower–Finisher Pigs Reared under Heat Stress
Source: Animals (Basel). 2023 Aug 19;13(16):2671. doi: 10.3390/ani13162671 (PMC10451215; doi:10.3390/ani13162671)
Supplement: Supplementary file 1 [file animals-13-02671-s001.zip › animals-2528490-SI.pdf]

animals

Table S1. Blood metabolites from pigs sampled at 47 d of experimental period.

	Treatments					
**Parameter**	**CONT[1]**	**RAC[2]**	**Met-Cr-S[3]**	**Met-Cr-L[4]**	**SEM[5]**	***p*-Value[6]**
Red blood cells, 1X10³ uL	5.18	5.17	5.11	4.94	0.074	0.648
Hemoglobin, g/dL	15.28	15.31	15.07	14.53	0.221	0.579
Hematrocrit, %	45.00	45.08	44.52	42.89	0.643	0.603
Leukocytes, 1X10³ uL	18.07	18.50	15.71	19.04	0.609	0.249
MCV[7]	86.88	87.20	87.11	86.80	0.090	0.365
MCH[8]	29.49	29.61	29.49	29.39	0.038	0.267
MCHC[9]	33.95	33.96	33.84	33.86	0.025	0.225
Lymphocytes, 1X10³ uL	11.01	10.41	8.71	10.41	0.399	0.227
Platelets, 1X10³ uL	171.20	184.80	153.00	178.90	13.377	0.849
Glucose, mg/dL	76.58	75.82	91.40	89.74	2.898	0.128
Total proteins, g/dL	7.24	7.17	7.10	7.05	0.111	0.933
Albumin, g/dL	2.95	3.01	2.84	2.83	0.049	0.508
Ck[10], u/L	93.06	158.26	109.99	166.36	10.546	0.198
Cortisol, µg/dL	3.96	2.76	4.79	3.14	0.326	0.151

[1] CONT: control animals receiving only the basal diet without supplementation; [2] RAC: animals receiving the basal diet supplemented with 10 ppm ractopamine during the last phase (34 d); [3] Met-Cr-S: animals receiving the basal diet supplemented with 0.8 ppm of Cr from chromium methionine only during the last phase for a short period (34 d); [4] Met-Cr-L: animals receiving the basal diet supplemented with 0.8 ppm of Cr from chromium methionine used in all phases for a long period (81 d); [5] SEM: standard error of the mean; [6] Probability values associated with the effects of chromium methionine or ractopamine supplementation in diets of pigs; [7] MCV: mean corpuscular volume; [8] MCH : mean corpuscular hemoglobin; [9] MCHC: mean corpuscular hemoglobin concentration; [10] CK: creatine kinase.

Table S2. Blood metabolites from pigs sampled at 81 d of feeding period

	Treatments					
**Parameter**	**CONT[1]**	**RAC[2]**	**Met-Cr-S[3]**	**Met-Cr-L[4]**	**SEM[5]**	***p*-Value[6]**
Red blood cells, 1X10^3 uL	5.25	5.36	5.29	5.05	0.063	0.365
Hemoglobin, g/dL	15.48	15.79	15.56	14.91	0.186	0.403
Hematrocrit, %	45.72	46.57	45.85	44.02	0.548	0.421
Leukocytes, 1X10^3 uLblan	16.90	16.88	15.02	16.24	0.561	0.610
MCV[7]	87.10	86.88	86.89	87.20	0.073	0.333
MCH[8]	29.49	29.45	29.40	29.52	0.024	0.323
MCHC[9]	33.85	33.90	33.86	33.87	0.010	0.333
Lymphocytes, 1X10^3 uL	9.67	8.69	7.71	9.42	0.440	0.409
Platelets, 1X10^3 uL	222.40	206.30	192.20	180.90	11.280	0.600
Glucose, mg/dL	84.98	86.08	78.70	86.81	2.085	0.512
Total proteins, g/dL	8.02^{ab}	8.59^{a}	8.07^{ab}	7.81^{b}	0.085	0.023
Albumin, g/dL	3.34^{b}	3.62^{a}	3.55^{ab}	3.39^{ab}	0.032	0.020
CK[10], u/L	29.79^{b}	78.25^{a}	36.44^{b}	37.67^{b}	4.659	0.004
Cortisol, µg/dL	2.47	2.42	2.42	3.15	0.270	0.727

[1] CONT: control animals receiving only the basal diet without supplementation; [2] RAC: animals receiving the basal diet supplemented with 10 ppm ractopamine during the last phase (34 d); [3] Met-Cr-S: animals receiving the basal diet supplemented with 0.8 ppm of Cr from chromium methionine only during the last phase for a short period (34 d); [4] Met-Cr-L: animals receiving the basal diet supplemented with 0.8 ppm of Cr from chromium methionine used in all phases for a long period (81 d); [5] SEM: standard error of the mean; [6] Probability values associated with the effects of chromium methionine or ractopamine supplementation in diets of pigs; [7] MCV: mean corpuscular volume; [8] MCH: mean corpuscular hemoglobin; [9] MCHC: mean corpuscular hemoglobin concentration; [10] CK: creatine kinase; [a,b] Within rows, means with different superscripts letter were significantly different ($P < 0.05$).

Table S3. Organ weight as a percentage of live weight (%).

	Treatments					
**Parameter**	**CONT[1]**	**RAC[2]**	**Met-Cr-S[3]**	**Met-Cr-L[4]**	**SEM[5]**	***p*-Value[6]**
Liver, %	1.43	1.44	1.46	1.54	0.025	0.429
Heart,%	0.34	0.32	0.31	0.31	0.006	0.369
Lungs,%	0.98	0.88	0.88	0.84	0.033	0.447
Stomach, %	0.53	0.51	0.53	0.54	0.012	0.809
Spleen, %	0.17	0.16	0.28	0.16	0.026	0.339
Kidneys, %	0.31	0.26	0.29	0.30	0.007	0.119

[1] CONT: control animals receiving only the basal diet without supplementation; [2] RAC: animals receiving the basal diet supplemented with 10 ppm ractopamine during the last phase (34 d); [3] Met-Cr-S: animals receiving the basal diet supplemented with 0.8 ppm of Cr from chromium methionine only during the last phase for a short period (34 d); [4] Met-Cr-L: animals receiving the basal diet supplemented with 0.8 ppm of Cr from chromium methionine used in all phases for a long period (81 d); [5] SEM: standard error of the mean; [6] Probability values associated with the effects of chromium methionine or ractopamine supplementation in diets of pigs.

Table S4. Proximate analysis and physicochemical characteristics of meat from pigs supplemented with chromium methionine or ractopamine under heat stress conditions.

	Treatments[1]					
**Variables**	**CONT[1]**	**RAC[2]**	**Met-Cr-S[3]**	**Met-Cr-L[4]**	**SEM[5]**	***p*-Value[6]**
Proximate Analysis						
Moisture, %	70.97	71.43	71.59	71.92	0.42	0.462
Fat, %	2.02	1.86	2.07	1.76	0.40	0.942
Protein crude, %	21.70	21.10	20.16	22.23	0.63	0.142
Ash; %	1.24	1.37	1.38	1.20	0.12	0.601
Physicochemical Analysis						
pH	5.48	5.43	5.46	5.47	5.02	0.905
WHC[7], %	78.34	78.69	79.35	77.04	1.44	0.720
WBSF[8], Kg	6.95	6.56	6.39	6.07	0.32	0.275
Cooking loss, %	21.71	22.92	21.52	21.67	1.15	0.812

[1] CONT: control animals receiving only the basal diet without supplementation; [2] RAC: animals receiving the basal diet supplemented with 10 ppm ractopamine during the last phase (34 d); [3] Met-Cr-S: animals receiving the basal diet supplemented with 0.8 ppm of Cr from chromium methionine only during the last phase for a short period (34 d); [4] Met-Cr-L: animals receiving the basal diet supplemented with 0.8 ppm of Cr from chromium methionine used in all phases for a long period (81 d);.[5] SEM: standard error of the mean. [6] Probability values associated with the effects of chromium methionine or ractopamine supplementation in the diets of pigs; [7] WHC: water holding capacity; [8] WBSF: warner–bratzler shear force.
